# Stretchable and Flexible High-Strain Sensors Made Using Carbon Nanotubes and Graphite Films on Natural Rubber

**DOI:** 10.3390/s140100868

**Published:** 2014-01-06

**Authors:** Sreenivasulu Tadakaluru, Wiradej Thongsuwan, Pisith Singjai

**Affiliations:** Materials Science Research Center, Department of Physics and Materials Science, Faculty of Science, Chiangmai University, Chiangmai 50200, Thailand; E-Mails: t.sreenivasulu87@gmail.com (S.T.); wiradej.t@cmu.ac.th (W.T.)

**Keywords:** piezoresistive sensor, soft wearable sensors, electro-mechanical properties, film composite, stretchable device, carbon nanotubes, health monitoring

## Abstract

Conventional metallic strain sensors are flexible, but they can sustain maximum strains of only ∼5%, so there is a need for sensors that can bear high strains for multifunctional applications. In this study, we report stretchable and flexible high-strain sensors that consist of entangled and randomly distributed multiwall carbon nanotubes or graphite flakes on a natural rubber substrate. Carbon nanotubes/graphite flakes were sandwiched in natural rubber to produce these high-strain sensors. Using field emission scanning electron microscopy, the morphology of the films for both the carbon nanotube and graphite sensors were assessed under different strain conditions (0% and 400% strain). As the strain was increased, the films fractured, resulting in an increase in the electrical resistance of the sensor; this change was reversible. Strains of up to 246% (graphite sensor) and 620% (carbon nanotube sensor) were measured; these values are respectively ∼50 and ∼120 times greater than those of conventional metallic strain sensors.

## 1. Introduction

Piezoresistive materials are materials in which the electrical resistance is a function of the internal strain [1]. Piezoresistive strain sensors can be grouped into two types; flexible, and flexible and stretchable sensors. Metallic strain sensors and polymer composite-based strain sensors are examples of flexible and flexible and stretchable strain sensors, respectively. Metallic strain sensors are widely used; the maximum strain measurable with these sensors is 5% [2]. There are a number of different types of polymer-based strain sensors [3]. They are typically made using conductive fillers such as single-walled carbon nanotubes, multiwalled carbon nanotubes (MWCNTs), carbon black, graphite in a polymer matrix, or film composites [4–6]. Polymer composite strain sensors offer a maximum measurable strain that is greater than that of metallic strain sensors. Sensors that can measure high strains typically show lower sensitivity, and highly sensitive sensors typically offer small maximum measurable strains [7,8]. Yamada *et al.* introduced a different method for the fabrication of strain sensors using aligned carbon nanotubes. These sensors could measure strains of up to 280% (sensitivity 0.82–0.06); the authors reported that maximum strains of only ∼5% were measurable using randomly aligned single wall carbon nanotubes [9]. Shin *et al.* reported a maximum measurable strain of up to 300% with a sensitivity 0.34–1.07 using a MWCNT forest [10]. Although these sensors' measurable strains were high, the sensitivity values were lower than those of commercially available metallic strain sensors, whose sensitivity typically has a value of 2.

Here, we report two types of strain sensors fabricated by sandwiching conductive films between natural rubber (NR) layers, where the conductive layers consisted of randomly distributed, entangled MWCNTs, or graphite flakes. These two types of sensors could measure high strains of up to 620% (MWCNT sensor) and 246% (graphite sensor), with high sensitivities of 5–43 and 12–346, respectively. The effect of the sample dimensions (width, length) on the sensitivity and growth rate of the relative resistance was also investigated. Most of the reported sensors' response curves are nonlinear, so the application of a linear fit leads to large errors. To solve this problem, we introduce a linearization method to linearize the exponential response curves.

## 2. Experimental Section

### 2.1. Fabrication of Multiwalled Carbon Nanotube/Graphite Flake Strain Sensors

MWCNTs were synthesized using the CVD method [11], and graphite powder was purchased from Merck Chemicals Darmstadt, Germany. These two carbon allotropes were chosen as conducting powders. The NR substrate (thickness 0.080 mm) was pre-stretched, and MWCNT/graphite powder was coated on the substrate by gently rubbing the powder on the surface by hand (MWCNT: ∼0.109 mg/cm^2^, graphite: ∼0.16 mg/cm^2^, thickness: ∼10–20 μm). The graphite/MWCNTs adhered well to the NR surface because of the surface stickiness of NR; this stickness included a dispersive adhesion mechanism as well as a chemical adhesion mechanism (no adhesives were used). This MWCNT-coated NR sheet was then cut into several samples.

**Figure 1.**
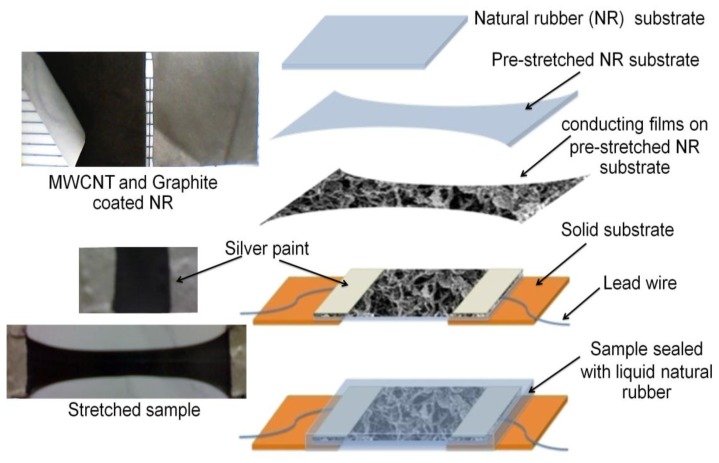
Schematic illustration of the steps used to prepare the test samples, which consisted of MWCNTs or graphite coated on NR.

**Figure 2.**
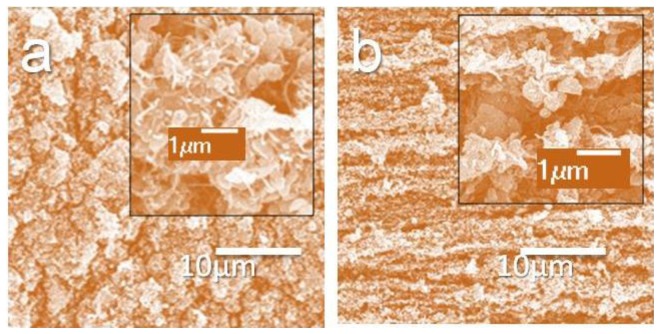

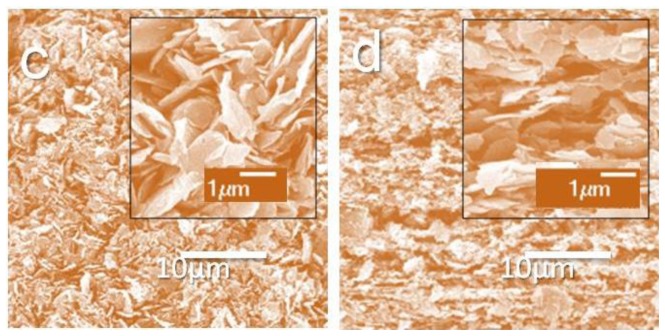
(**a,b**) SEM images of unstrained (0%) and strained (400%) MWCNT-coated NR samples; (**c,d**) SEM images of unstrained (0%) and strained (400%) graphite-coated NR samples.

In the experiments, the two ends of the MWCNT-coated NR sample were strongly glued on two supporting acrylic pieces. Two electric wires were attached at the ends of the sample, using silver paint (on the strongly glued area of the sample). Because the silver paint was on the strongly glued, unstretchable area of the sample, no cracking of the silver paint occurred during the stretching of the sample. After the preparation of the electrodes, the sample was sealed with liquid natural rubber and dried. This sandwiched, middle layer of MWCNTs between the two NR layers acted as a strain sensor. The graphite sensors were prepared in the same way as the MWCNT sensors. A schematic diagram of the key sample fabrication steps is shown in Figure 1, and the surface morphologies (unsealed sample) of 0%- and 400%-strained MWCNT and graphite samples are shown in Figure 2. The FE-SEM micrographs showed that even at high strains, there was still a conducting path between the nanotubes, and between the graphite flakes. These results suggested that the sensors would have a measurable strain range.

## 3. Results and Discussion

### 3.1. Electrical Resistance Measurements

**Figure 3.**
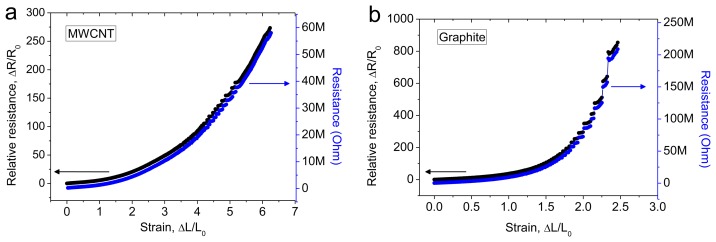
Response curves: (**a**) Relative resistance and resistance *versus* strain response curves for the MWCNT sensor; (**b**) Relative resistance and resistance *versus* strain response curves for the graphite sensor.

The MWCNT sandwich sample (gauge length 2 cm, width 1 cm) was stretched using a constant strain rate of 8.3% s^−1^, up to a strain of 620% (giving a final sample length of 14.4 cm). For the graphite flake sandwich sample (active gauge length 2 cm, width 1 cm), the maximum measured strain was 246% (giving a final sample length of 6.92 cm). The electrical resistance was measured by using Kelvin (4-wire) method. The resistance and relative change in resistance *versus* strain response curves are shown in Figure 3 for the MWCNT and graphite samples.

The resistance increased with the strain [1,4,5,7]. The carbon nanotube sensor showed a higher measurable strain limit (620%) compared with the graphite sensor (246%); this was likely because the nanotubes had a smaller size, compared with the graphite flakes. The sensitivity was calculated in terms of the gauge factor, using the formula gauge factor (GF) = relative resistance/strain, *i.e.*, GF = [(R − R_0_)/R_0_]/[(L − L_0_)/L_0_], where R_0_ and L_0_ are the initial resistance and initial length of the sample, respectively. The sensitivity increased linearly with increasing applied strain for the MWCNT sensor, and the sensitivity increased exponentially with increasing applied strain for the graphite sensor. The sensitivity values for the MWCNT sensors were 5.5, 10.2, 16.3, 23.1, 31.5, and 43.4 at 100%, 200%, 300%, 400%, 500%, and 620% strain, respectively. The sensitivity values for the graphite sensors were 23.7, 37.5, 148.1, and 346.6 at 50%, 100%, 200%, and 246% strain, respectively.

**Figure 4.**
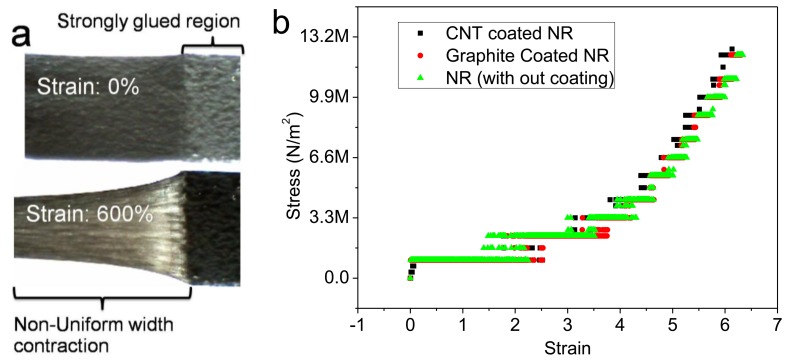
Nonlinearity: (**a**) Non-uniform deformation of the strained conducting layer/substrate; (**b**) Stress *versus* strain curves for the MWCNT, graphite coated, and uncoated NR samples.

The relative change in the resistance response curves for the MWCNT and graphite sensors was nonlinear. It is likely that this nonlinear behavior resulted from the non-uniform fracturing/deformation of the conducting layers on the NR. When the sensor was subjected to stretch, the width contracted perpendicular to the longitudinal strain axis. During stretching, the freedom of the polymer chains to undergo contraction (perpendicular to the longitudinal strain axis) decreased exponentially with distance from the ends. This width contraction (or the lateral strain), was much lower at the sample's two ends, compared with the middle of the sample. This was because the sample ends were strongly bonded to the acrylic holding substrate with super-glue (Figure 4a). The stress *versus* strain curves for the MWCNT, graphite coated, and uncoated NR samples were identical and nonlinear (*i.e.*, they did not obey Hook's law) (Figure 4b). At the ends of the sample where the polymer chains had less freedom to undergo contraction, the level of fracturing in the conducting layer was higher. This resulted in a reduction in the electrical percolation/number of tunneling contacts with increasing strain. The tunneling resistance may therefore have increased with the decrease in the electrical percolation/number of tunneling contacts [12–14].

### 3.2. Effect of Sensor Size on Linearity and Sensitivity

**Figure 5.**
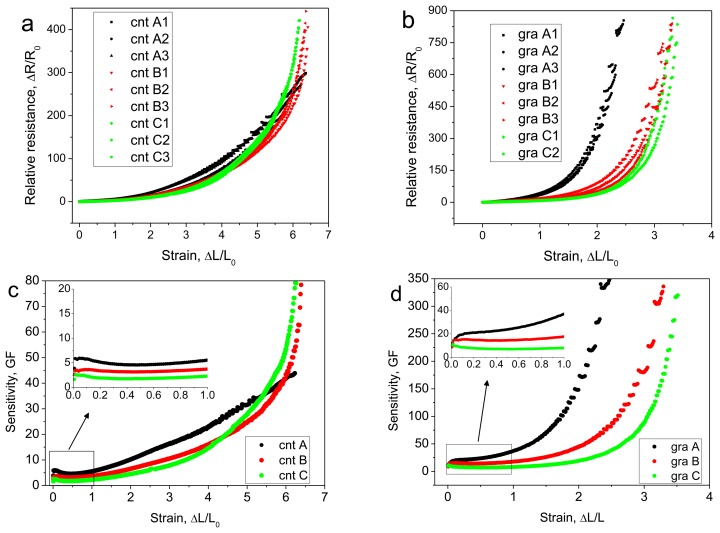
Size dependent sensitivity: (**a**) MWCNT sensors with dimensions (width × length) of 10 × 20 mm (A1, A2, A3); 10 × 10 mm (B1, B2, B3); and 10 × 5 mm (C1, C2, C3); (**b**) Graphite sensors with dimensions (width × length) of 10 × 20 mm (A1, A2, A3); 10 × 10 mm (B1, B2, B3); 10 × 5 mm (C1, C2); (**c**,**d**) Sensitivity *versus* strain curves for MWCNT and graphite sensors with dimensions of 10 × 20 mm (A), 10 × 10 mm (B), and 10 × 5 mm (C).

MWCNT and graphite sensors were prepared with a constant width of 10 mm, and three different lengths (active gauge lengths of (A) 20 mm; (B) 10 mm; and (C) 5 mm were prepared). The three samples A, B, and C were stretched using a constant strain rate of ∼8% s^−1^, and the electrical resistance was recorded. The longest MWCNT sensors and graphite sensors (10 × 20 mm) showed higher sensitivity than the shorter samples in the lower strain range. The shorter samples' response curves were highly nonlinear compared with the long samples, and at higher strain values the sensitivities of the shorter MWCNT samples surpassed the long samples' sensitivity. The measured relative resistance growth rate for the MWCNT sensors using sample sizes of W × L = 10 × 20 mm, 10 × 10 mm, and 10 × 5 mm were 0.548, 0.773, and 0.870, and those for the graphite sensors were 2.262, 2.298, and 2.430. The relative change in resistance *versus* strain and the sensitivity *versus* strain are shown in Figure 5 for the three different samples (A, B, and C).

For typical metallic strain sensors, the gauge factor value is constant (∼2) up to the maximum strain limit of 5%. Slobodian *et al.* [15] reported a sensor that could measure strains of up to 400% (MWCNT network in polyurethane); even at low strain values, its sensitivity changed linearly. Shin *et al.* reported a sensor (MWCNT forest in polyurethane) that showed constant sensitivity (0.34) up to a 30% strain limit. Yamada *et al.* reported constant sensitivity (0.82) up to a 40% strain limit. In the present work, the MWCNT/graphite sensor showed a constant sensitivity (5/12) for strain values of up to 100%.

### 3.3. Multiple Cycle Tests

**Figure 6.**
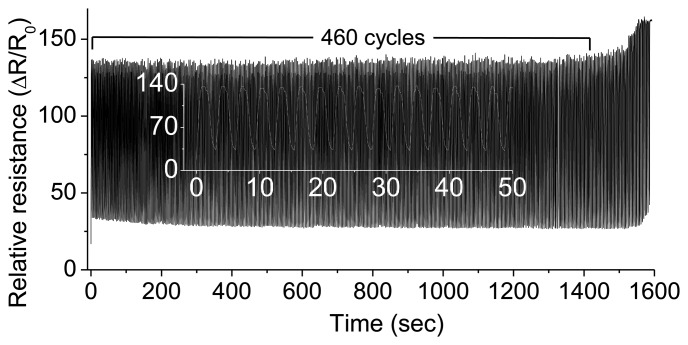
Multiple cycle tests: More than 400 cycles of period 3.04 s were carried out for the strain range of 150%–500%. (Inset image: the first 16 full cycles carried out over a time period of 48.6 s).

One end of the MWCNT strain sensor sample was attached to a disk, which was rotating with an angular velocity of 2 radians·s^−1^. The applied sinusoidal strain (with a period of ∼3.04 s) ranged between 150% and 500%, and the total number of cycles was more than 400 (Figure 6).

### 3.4. Linearization of Nonlinear Response Curves

**Figure 7.**
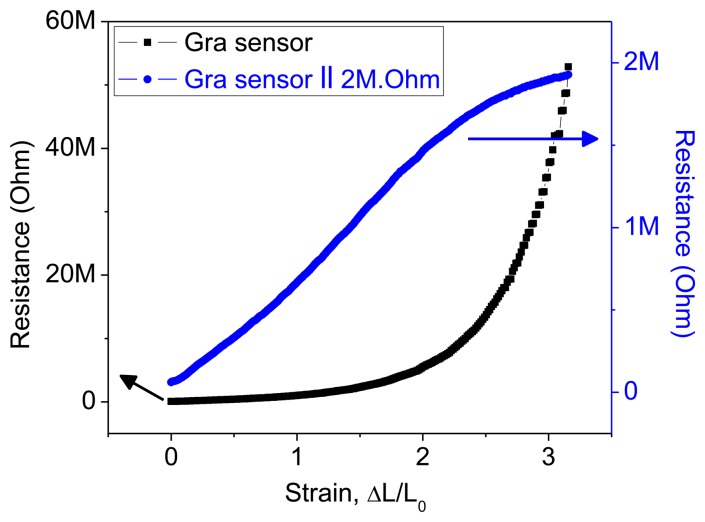
Linearization: response curves for graphite sensor with (blue) and without (black) a 2 MΩ parallel resistor.

Metallic strain sensors' response curves are linear up to ∼5% of the maximum strain limit; for small strain values, therefore, the response curve for the graphite sensor appeared to be linear. However, the response curve over the whole region from 0% to 300% strain showed that this linearity was merely an approximation to the early part of an exponential curve. The linear response curve of conventional metallic strain sensors can therefore be considered as a small part of an exponential curve. A linear fit to an exponential response curve results in large errors. However, these exponential response curves could be linearized by connecting an external resistor parallel to the strain sensor. Resistance curves for the graphite sensor with and without a 2 MΩ parallel resistance are shown in Figure 7.

The coating of the MWCNT/graphite flakes on the prestretched substrate helped to maintain the electrical percolation and conducting pathways between the two electrodes (electric leads), even at high strains. Axial strain was applied using a lab-made speed control device. The two electrical leads from the test sample were connected to a constant power supply of 5 V (laboratory D.C power supply, GPC-1850D, Tucheng, New Taipei, Taiwan). The current flow in the circuit was measured using a Keithley-196 system DMM instrument, Cleaveland, Ohio, USA. The voltage drop across the sensor was measured (Kelvin method or 4-wire method) using a Hewlett Packard data acquisition-34970A (Santa Clara, CA, USA) instrument, and the output resistance was recorded using a computer. The surface morphology of the strained films was observed using FE-SEM (JSM-6335F, JEOL, Akishima, Tokyo, Japan).

## 4. Conclusions

To the best of our knowledge, these results are superior to those of any other previously reported strain sensor, in terms of the simple fabrication, high measurable strain (620%, limited by the substrate properties), and high sensitivity with good durability (400 cycles for 150%–500% strain range). These high-strain sensors could be used as multifunctional sensors [16] to measure, for example, force, speed, pressure, acceleration, frequency, structural vibrations, earthquakes or in health monitoring. The NR substrate has characteristic limitations that prevent its use in some environments, but these limitations could be overcome by replacing the NR substrate with other types of elastomers, for example, silicone rubber.

## Supplementary Material

**Supplementary Information:** Response curves of MWCNT and Graphite sensors for different samples/sensors sizes (Figure S1), cross sectional views of MWCNT and Graphite films on natural rubber (Figure S2) and comparison with other reported results (Table S1) were shown in supplementary information.
