# Supplementary material for: Stretchable and Flexible High-Strain Sensors Made Using Carbon Nanotubes and Graphite Films on Natural Rubber
Source: Sensors (Basel). 2014 Jan 6;14(1):868–76. doi: 10.3390/s140100868 (PMC3926590; doi:10.3390/s140100868)
Supplement: Supplementary file 1 — Supplementary Information: Response curves of MWCNT and Graphite sensors for different samples/sensors sizes (Figure S1), cross sectional views of MWCNT and Graphite films on natural rubber (Figure S2) and comparison with other reported results (Table S1) were shown in supplementary information. [file sensors-14-00868-s001.pdf]

OPEN ACCESS
sensors
ISSN 1424-8220
www.mdpi.com/journal/sensors

Supplementary Information

Stretchable and Flexible High-Strain Sensors Made Using Carbon Nanotubes and Graphite Films on Natural Rubber. *Sensors* 2014, *14*, 868-876

Sreenivasulu Tadakaluru, Wiradej Thongsuwan and Pisith Singjai *

Materials Science Research Center, Department of Physics and Materials Science, Faculty of Science, Chiangmai University, Chiangmai 50200, Thailand; E-Mails: t.sreenivasulu87@gmail.com (S.T.); wiradej.t@cmu.ac.th (W.T.)

* Author to whom correspondence should be addressed; E-Mail: pisith.s@cmu.ac.th; Tel.: +66-538-922-71; Fax: +66-538-922-70.

Figure S1. Effect of sample size on sensitivity and linearity: Response curves of MWCNT (Cnt) and Graphite (Gra) sensors for different samples/sensor sizes [W × L = 10 × 20 mm (A1, A2, A3), 10 × 10 mm (B1, B2, B3), 10 × 5 mm (C1, C2, C3)].

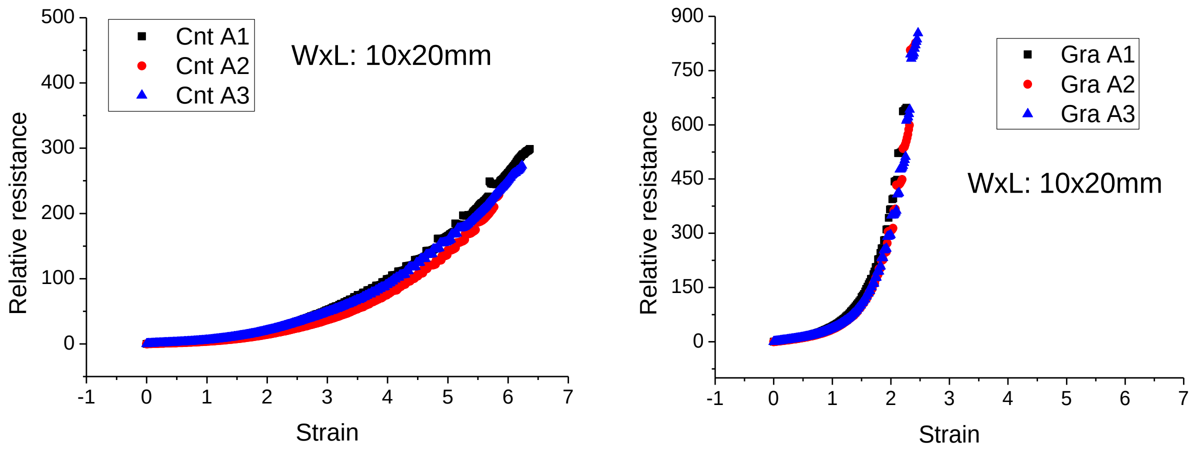

Figure S1. *Cont.*

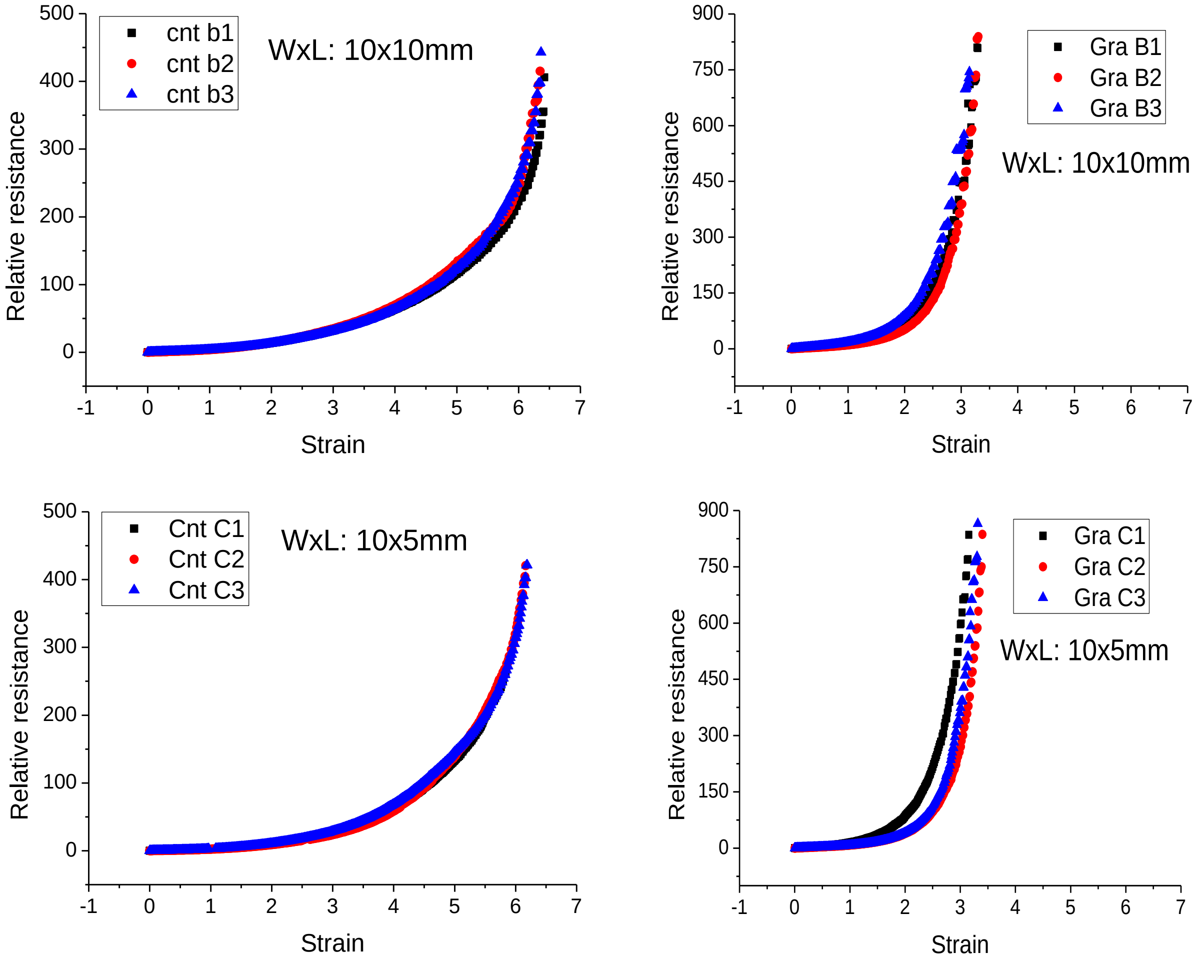

Figure S2. Cross sectional views (**a**) MWCNT and (**b**) Graphite films (scale value is 10 μm).

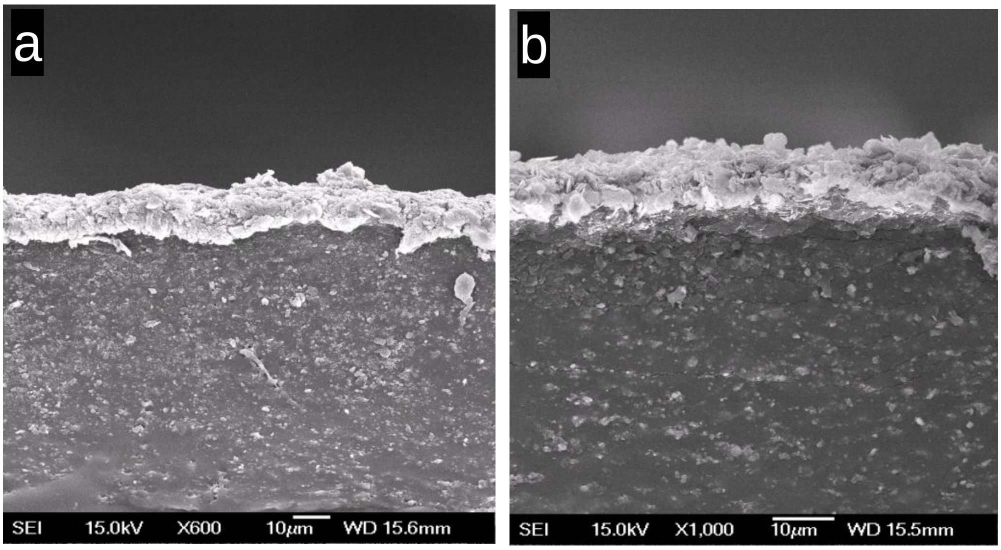

Table S1. Comparison with other reported results.

Sensor/Device [Ref]	Max. Strain (%)	Response Curve Type	Sensitivity	Sensitivity Relation with Strain	Durability and Repeatability	Delay Time (ms)
(a) Random MWCNT	620 (Limited by the substrate elongation limit)	Close to linear	5–43	Linearly Increased with strain	>400 cycles at 150%–500% strain (strain rate ~115% s^{-1})	
(b) Graphite Film sandwich in NR [present work]	246	Exponential	12–346	Increased with strain		
(a) Aligned SWCNT	280	Two linear regions	0.82–0.06	Increased with strain	~3,300 cycles at 200% strain	14 @ 2.5 Hz
(b) Random SWCNT film on PDMS [9]	<6	-	-		-	-
MWCNT forest/PU composite [10]	300	Close to linear	0.34–1.07	Increased with strain	>100 cycles at 80% strain	-
MWCNT network/PU [15]	403	Close to linear	4–69	Linearly Increased with strain	>1000 cycles at <100% strain	-
PEO-MWCNT composite [6]	<0.4	Close to linear	~6.0–12.5	Increased with strain	-	-
MWCNT Bucky paper sandwich [7]	<0.2	-	2.62 & 38.2 (4 point probe)	Increased with strain	-	-
EVA-Graphite composite [1]	~10	Exponential	~1600 (at 10% strain)	Increased with strain	-	-

© 2014 by the authors; licensee MDPI, Basel, Switzerland. This article is an open access article distributed under the terms and conditions of the Creative Commons Attribution license (http://creativecommons.org/licenses/by/3.0/).
